# The Social and Financial Burden on Families of Type 1 Diabetic Pediatric Patients in Madinah Region, Saudi Arabia

**DOI:** 10.7759/cureus.66427

**Published:** 2024-08-08

**Authors:** Abdulaziz Marwi, Ibrahim A Bali, Abdulhalim Almurashi, Eman H Alharbi, Ibtesam J Alnkhli, Nader Moneer Alqerafi

**Affiliations:** 1 Preventive Medicine, Public Health Administration, Ministry of Health, Madinah, SAU; 2 Pediatric Endocrinology, King Salman Bin Abdulaziz Medical City, Madinah, SAU; 3 Diabetes Center, King Fahd Hospital, Madinah, SAU; 4 Pediatric Medicine, King Salman Bin Abdulaziz Medical City, Madinah, SAU; 5 Nursing, King Salman Bin Abdulaziz Medical City, Madinah, SAU; 6 Health Affairs, Ministry of Health, Madinah, SAU

**Keywords:** saudi arabia, pediatric patients, caregivers’ quality of life, caregiver burden, type 1 diabetes mellitus

## Abstract

Introduction: Caregivers and families play an essential role in managing and caring for type 1 diabetes mellitus (T1DM) pediatric patients. However, caregiving is usually associated with social and financial burdens. This study assesses the burden and underlying social and financial factors among Saudi caregivers of pediatric patients with T1DM.

Methods: A cross-sectional study was conducted among caregivers and families of T1DM pediatric patients attending the Diabetic Center at King Fahad Hospital (KFH) and the Diabetic Center at King Salman Bin Abdulaziz Medical City (KSAMC) in Al-Madinah City, Kingdom of Saudi Arabia (KSA) from January 2024 to June 2024. The data collection was done using the Zarit Burden Questionnaire and the Caregiver Care Cost Assessment Questionnaire.

Results: The study surveyed 376 participants, primarily females (N = 285, 75.8%) and married (N = 317, 84.3%), with a majority aged between 18 and 47 years (N = 322, 85.6%). The burden experienced by families with T1DM pediatric patients was moderate, with a mean total burden level score of 27.8 ± 16.3. For those employed, most reported working as usual (N = 107, 81.1%), with most spending less than two hours on care weekly (N = 76, 57.6%). A significant association was found between the financial impact of caregiving on saving, spending, and general financial stress and social burden (p < 0.01).

Conclusion: The findings show a moderate burden faced by caretakers of T1DM pediatric patients, with a strong correlation between the financial impact of caregiving on saving, spending, and general financial stress and burden level. The findings also highlight the significant impact of caregiving on the financial stress and lifestyle changes that caregivers endure.

## Introduction

Type 1 diabetes mellitus (T1DM) is a chronic autoimmune condition where the immune system attacks the insulin-producing beta cells in the pancreas, resulting in the body producing very little or no insulin [1]. An estimated 8.4 million people around the world had T1DM in 2021, and this number may rise to 17.4 million by 2040 [2]. Among children and adolescents, the annual global increase is estimated at around 3%, although there are significant geographical variations. While incidence is greatest in northern European countries, including Finland and Sweden, the Kingdom of Saudi Arabia (KSA) has the eighth highest incidence rate [2,3]. Approximately 5-10% of all diabetic patients in the KSA are T1DM patients [1]. The high rates of disability and mortality associated with T1DM cause a great burden to patients, their families, and society [2,3]. The burden includes disruption in the caregiver’s domestic routine and social activities, financial loss, and loss of productive hours [4]. It may also include the subjective feelings of distress, grief, and worry, which may be present in the caregiver [5]. These difficulties further lead to significant changes in patients’ lifestyles, causing emotional distress. In fact, patients with T1DM are twice as likely to suffer from anxiety and depression than those without diabetes [2]. At home, the treatment of T1DM often requires the involvement of family members. Family or informal caregivers are often offspring or spouses that provide unpaid support, with an important role in monitoring patients’ self-management, detecting improvements or deteriorations in the disease progression, as well as in providing daily care [6]. Although family caregivers often feel unprepared to provide care, they accept their role mostly because of feelings of moral or social obligation.

It is important to identify what support patients with T1DM may need to deal with the stress involved with this serious condition, and its impact on quality of life (QoL). Psychological interventions, such as relaxation training techniques or hypnosis, have already shown positive results in the management of T1DM [7,8]. Financial difficulties and a lack of universal health services can also impact a family’s ability to provide adequate care to the extent that some children suffer complications, including seizures and coma [9]. To our knowledge, no study has been conducted in Al-Madinah city to address this issue. Therefore, this study aimed to assess the level of burden, and underlying social and financial factors among caregivers of patients with T1DM.

## Materials and methods

Study setting and population

This was an analytical cross-sectional study conducted from January 2024 to June 2024 in Al-Madinah City. Al-Madinah has a population of about two million people, and Diabetic Centers at King Fahad Hospital (KFH) and King Salman Bin Abdulaziz Medical City (KSAMC) are the main institutes with care services for diabetic patients.

This study was conducted among Saudi caregivers or families of type 1 diabetic pediatric patients aged 18 years and younger in Al-Madinah City, KSA. We excluded caregivers of patients with type 2 diabetes mellitus, patients aged more than 18 years, and patients with concomitant diseases with non-Saudi nationality. Based on approximately 2000 T1DM patients in the Al-Madinah region, the target sample size calculated using the Epi Info sample size calculator (Centers for Disease Control and Prevention, Atlanta, GA) was 323, with a 5% margin error and a 95% confidence interval. Adding 10% to compensate for possible non-response, the final sample size was 355.

A simple random selection technique was used to select eligible participants from the two main healthcare facilities managing T1DM pediatric patients in Al-Madinah City (KFH and KSAMC Diabetic Centers).

Data collection tool

We used a pre-validated questionnaire with 22 items, the Zarit Burden Questionnaire [10], to assess the level of social burden experienced by caregivers of patients with T1DM. The questionnaire explores the negative mental, physical, social, and economic impacts on the lives of caregivers. It is scored using a five-point Likert scale with responses ranging from 0 (never) to 4 (nearly always) and scores caregivers on a total score of 88 [10]. For this study, the scores were further dichotomized into low burden (0-40) and high burden (41-88). To assess the care costs to caregivers, we used the Caregiver Care Cost Assessment Questionnaire with 13 questions concerning caregiver work status and the provision of paid and unpaid informal care [11]. Different steps are involved in estimating costs, such as indirect (productivity) and informal care costs of illness, based on the data collected using the questionnaire. Indirect cost estimation encompasses six steps, differentiating between caregivers who are employed full-time, employed part-time, and unemployed. Estimating informal care costs includes three steps, differentiating between paid and unpaid informal care.

The questionnaire was piloted in 10% of the sample size to identify difficulties and time required to finish the questionnaire. Then, an epidemiologist reviewed the questionnaire to assess its methodological quality. Participants of the pilot study were excluded from the main study.

Data collection technique

The investigator described the aim and objectives of the study to participants and asked them to provide consent after they received all the needed information. Questionnaires were printed and distributed to caregivers of T1DM pediatric patients in Al-Madinah. The responses were collected and entered into an Excel sheet (Microsoft Corporation, Redmond, WA).

Study variables

The outcome variable was the level of social and financial burden of families of T1DM patients in Al-Madinah, KSA, while independent variables included demographic characteristics, such as age, gender, relation to the patient, financial status, educational level, and social status.

Data analysis

The data were analyzed using the SPSS version 24 (IBM Corp., Armonk, NY). Descriptive statistics were used to summarize the data. Continuous variables were expressed as mean and standard deviation, while frequencies of categorical variables were expressed as percentages. The chi-square tests were used to analyze categorical variables. Correlation analysis was used to identify predictors of burden of care. The significance level was set at p < 0.05, and confidence intervals were calculated.

Ethical considerations

All participants’ information was kept confidential and used only in the study research process. The investigator described the aim and objectives of the study to participants and asked them to provide consent after they received all the needed information. This study was approved by the Institutional Review Board (IRB), General Directorate of Health Affairs in Madinah (IRB log No.: 24-022).

## Results

This study received 376 responses from a total of 392 participants (95.92% response rate), and Table 1 shows the demographic characteristics of the study sample. The majority of participants were female (N = 285, 75.8%), and most were married (N = 317, 84.3%). The age distribution shows a large number in the 18-47-year range: 184 (48.9%) were aged from 18 to 38 years and 136 (36.7%) were aged from 39 to 47 years. Regarding monthly income, a considerable proportion (N = 273, 72.6%) had an income of less than 10000 Saudi Riyal (SAR), while 16.0% earned between 10000 and 14999 SAR. Educational levels were diverse; the highest percentage (N = 189, 50.3%) held a bachelor’s or higher degree, followed by 115 (30.6%) with a secondary education.

**Table 1:** Demographic characteristics of the study sample.

Variables		Frequency	Percent
Gender	Female	285	75.8
	Male	91	24.2
Marital status	Divorced or widower	28	7.4
	Single	31	8.2
	Married	317	84.3
Age in years	18-38	184	48.9
	39-47	138	36.7
	48-58	54	14.4
Monthly income (Saudi Riyal)	<10000	273	72.6
	10000-14999	60	16.0
	>15000	43	11.4
Educational levels	Primary	27	7.2
	Middle	45	12.0
	Secondary	115	30.6
	University or higher	189	50.3

Table 2 presents the data about the burden experienced by families with T1DM pediatric patients. The results show that most participants (N = 290, 77.1%) reported a low burden, while 86 (22.9%) experienced a high burden. The mean total burden level score was 27.8 ± 16.3 (out of a possible maximum score of 88), indicating a moderate overall burden level.

**Table 2:** The level of social burden distribution in the study.

Level of burden	Frequency	Percent
Low burden	290	77.1%
High burden	86	22.9%
Total burden level score	Mean ± SD	27.8 ± 16.3

Table 3 presents a comparison of findings of the burden levels experienced by families based on various demographic variables. No significant differences in burden levels were observed based on gender, marital status, age, monthly income, or educational level (p > 0.05).

**Table 3:** Comparison of burden levels among participants in the study. Values represent numbers (percentages). The p-value is calculated using the chi-square test.

Variables		Low burden	High burden	P-value
Gender	Female	218 (76.5%)	67 (23.5%)	0.603
	Male	72 (79.1%)	19 (20.9%)	
Marital status	Divorced or widower	24 (85.7%)	4 (14.3%)	0.398
	Single	22 (71.0%)	9 (29.0%)	
	Married	244 (77.0%)	73 (23.0%)	
Age in years	18-38	139 (75.5%)	45 (24.5%)	0.173
	39-47	104 (75.4%)	34 (26.4%)	
	48-58	47 (87.0%)	7 (13.0%)	
Monthly income (Saudi Riyal)	<10000	216 (79.1%)	57 (20.9%)	0.399
	10000-14999	43 (72.9%)	16 (27.1%)	
	>15000	31 (74.1%)	12 (25.9%)	
Educational levels	Primary	20 (75.9%)	7 (24.1%)	0.206
	Middle	37 (82.2%)	8 (17.8%)	
	Secondary	95 (82.6%)	20 (17.4%)	
	University or higher	138 (73.0%)	51 (27.0%)	

Tables 4-6 show the findings about the impact of caregiving, and the majority of the respondents were not employed (N = 244, 64.9%). Most respondents worked five days a week (N = 115, 63.2%), and did not reduce their working days due to a relative's condition (N = 161, 75.9%). For those who did reduce their working days, most still worked five days a week (N = 85, 59.0%). Concerning the impact on productivity last week, most respondents reported working as usual (N = 116, 56.9%). Also, the majority had not stopped working due to a relative's condition (N = 274, 81.1%), and most spent less than two hours on care weekly (N = 151, 44.7%). There are notable financial stresses and lifestyle changes reported by respondents, with many indicating that they have had to stop saving money, take loans, and reduce spending on non-essential items.

**Table 4:** Impact of caregiving on employment and daily activities.

Items		Frequency	Percent
Employment status	Yes	132	35.1
	No	244	64.9
	Total	376	100.0
Working days per week	Two days a week	1	0.8
	Three days a week	1	0.8
	Four days a week	2	1.1
	Five days a week	100	75.8
	Six days a week	12	9.1
	All days of the week	17	12.9
	Total	132	100.0
Reduced working days due to relative's condition	No	103	78.0
	Yes	29	22.0
	Total	132	100.0
Number of working days after reduction	One day a week	6.0	4.5
	Two days a week	4.0	3.0
	Three days a week	5.0	3.8
	Four days a week	20.0	15.2
	Five days a week	84.0	63.6
	Six days a week	13.0	9.8
	Total	132	100.0
Stopped working due to relative's condition	No	113	85.6
	Yes	19	14.4
	Total	132	100.0
Hours spent on care weekly	Less than two hours	68	51.5
	From two to 4 hours	36	27.3
	More than 4 hours	28	21.2
	Total	132	100.0
Additional hours spent on care weekly	Less than two hours	76	57.6
	From two to 4 hours	28	19.7
	More than 4 hours	28	19.7
	Total	132	100.0

**Table 5:** Impact on productivity and absenteeism due to caregiving.

Items		Frequency	Percent
Impact on productivity last week	Working as usual	107	81.1
	Moderate impact	22	16.7
	Could not work at all	3	2.3
	Total	132	100.0
Number of days absent last week	0	94	71.1
	1	11	8.3
	2	13	9.8
	3	3	2.3
	4	5	3.8
	5	1	0.8
	6	1	0.8
	7	4	3.0
	Total	132	100.0

**Table 6:** Financial impact of caregiving on saving, spending, and general financial stress.

Items		Frequency	Percent
Stopped saving money	No	67	50.8
	Yes	65	49.2
Taking a loan	No	79	59.8
	Yes	53	40.2
Unable to cover for essential needs	No	85	64.4
	Yes	47	35.6
Stopped spending money on clothing and dining as usual because of caregiving	No	63	47.7
	Yes	69	52.3
Stopped leisure activities as a consequence of caregiving	No	61	46.2
	Yes	71	53.8
Stressed financially in general because of caregiving	No	60	45.5
	Yes	72	54.5

In addition, Table 7 shows a significant association between the financial impact of caregiving and the social burden experienced by participants in the study, as evidenced by their saving, spending, and general financial stress behaviors. A significantly higher percentage of individuals in the high-burden category reported ceasing to save money compared to those in the low-burden group (p = 0.003). Similarly, taking out loans was significantly more common among those with a high burden than those with a low burden (p < 0.001). The inability to cover essential needs was significantly reported more by the high-burden group than the low-burden group (p < 0.001). Furthermore, significant differences were observed in spending behavior, with more high-burden individuals having stopped spending on clothing and dining out as usual than the low-burden group (p < 0.001). Leisure activities were also significantly affected, mostly in the high-burden group who ceased leisure activities, compared to the low-burden group (p < 0.001). Overall, the high-burden group reported significantly higher general financial stress due to caregiving, compared to the low-burden group (p < 0.001). These findings highlight the substantial financial and social burdens faced by caregivers, underscoring the need for targeted support and interventions.

**Table 7:** Association between the financial impact of caregiving on saving, spending, and general financial stress and social burden. The p-value is calculated using the chi-square test. ** Significant at < 0.01.

Items		High burden	Low burden	P-value
Stopped saving money	No	9 (13.4%)	58 (86.6%)	0.003**
	Yes	23 (35.4%)	42 (64.6%)	
Taking a loan	No	8 (10.1%)	71 (89.9%)	<0.001**
	Yes	24 (45.3%)	29 (54.7%)	
Unable to cover for essential needs	No	9 (10.6%)	76 (89.4%)	<0.001**
	Yes	23 (48.9%)	24 (51.1%)	
Stopped spending money on clothing and dining as usual because of caregiving	No	2 (3.2%)	61 (96.8%)	<0.001**
	Yes	30 (43.5%)	39 (56.5%)	
Stopped leisure activities as a consequence of caregiving	No	2 (3.3%)	59 (96.7%)	<0.001**
	Yes	30 (42.3%)	41 (57.7%)	
Stressed financially in general because of caregiving	No	3 (5.0%)	57 (95.0%)	<0.001**
	Yes	29 (40.3%)	43 (59.7%)	

## Discussion

The study aimed to assess the burden and psychological distress and underlying social and financial factors among Saudi caregivers of patients with T1DM. The results illustrated multifaceted caregiver burden dimensions, including financial, physical, social, spiritual, and emotional or mental stresses. This study is significant because it provides insights into what caregivers’ experiences entail as they care for children with T1DM, thus informing efforts at alleviating care burdens and enhancing their quality of life.

We found that the majority (N = 190, 77.1%) of respondents reported a low burden, while 86 (22.9%) reported a high burden. The mean total score for burden level was 27.8 ± 16.3, indicating moderate overall burden level experienced by the respondents. These findings are in line with similar studies that have shown how caregiving affects both physical and mental health for caregivers [12,13]. A study assessing the burden of care and psychological distress among primary caregivers of patients with type 2 diabetes mellitus (T2DM) found that caregivers had different levels of stress, with more females experiencing high stress than males [14]. Additionally, another study showed that anxiety disorders, depression symptoms, and fear from hypoglycemia episodes are common among caregivers of T1DM pediatric patients, along with social dysfunction due to severe diseases of their patients [15]. This indicated the need for social support for both patients and their caregivers. This is confirmed by other studies that have revealed how important social support is for caregiver’s mental health, suggesting that providing social support programs and respite care would substantially reduce caregiver burden while improving patients’ and caregivers’ lives [13-15]. Though 285 (75.8%) of our participants were female, which is consistent with women dominating caregiving services [16], our study did not indicate any statistically significant contribution of gender to the burden of care (p = 0.803). Further studies contrasted our findings by showing that female caregivers experience more stress and strain than male caregivers [13,17]. This study also did not find a statistically significant association between age and burden level. This conflict is compared with previous studies assessing the effect of caregiving on mental and physical health among the younger population of caregivers [14,18]. This negative impact of caregiving on mental and physical health, particularly among young caregivers and patients, indicates the need for targeted interventions, such as psychological interventions coupled with supportive services, to assist the caregivers and patients in bearing the burdens.

This study sheds light on financial difficulties caregivers encounter, such as stopping savings, taking loans, and not buying non-essential commodities, in addition to a strong positive correlation between stopping leisure activities and financial stress related to caregiving. This is consistent with several other studies pointing out the economic burdens related to caregiving, such as adjustment of work schedules and reduction in spending on leisure activities [15,18]. Our findings highlighted a positive correlation between stopping saving money and financial stress due to caregiving. This would imply that those who are no longer saving money tend to become more financially burdened with caregiving duties. This might explain a significant positive correlation between taking a loan and feeling overwhelmed financially due to providing care, which means that the financial distress revolves around failure to meet basic needs. Previous studies also found a significant impact of financial stress on caregivers’ mental and physical health [14,15,18], suggesting that financial aid and support programs might reduce caregiver burden and enhance the quality of life for care recipients as well as their caregivers.

The findings of this study have important implications for healthcare providers and policymakers. They underscore the necessity of comprehensive support systems addressing caregivers’ financial, emotional, and psychosocial concerns. This may involve financial aid, respite care, or even social support meant to help caregivers cope with caregiving burdens. However, this study has some limitations to consider. This study centered solely on caregivers and did not include patients’ perspectives. This can lead to a skewed understanding of the issues at hand, as caregivers and patients often have different experiences, needs, and insights regarding care. This may lead to biased care strategies. Future research should include patient feedback, use a mixed-methods approach, and conduct longitudinal studies to gain a holistic view of caregiving experiences. The study's cross-sectional design is limited in establishing causal relationships. Future longitudinal studies should explore further the burden of caregiving to T1DM pediatric patients.

## Conclusions

This study provides insights into what caregivers undergo while taking care of T1DM patients. The findings show a moderate burden faced by caretakers of T1DM patients, with a strong correlation between age and burden level. The findings also highlight the significant financial stress and lifestyle changes that caregivers endure, notably in terms of saving money, taking out loans, and cutting back on non-essential purchases and leisure costs. The strong correlations between financial stress and numerous aspects of caregiving highlight the significance of addressing the financial repercussions of caring for a patient with T1DM. This study’s findings suggest that there should be holistic support mechanisms to lessen care burdens. Further, longitudinal studies need to explore the caregiving burden involving caregivers and patients to develop successful interventions to improve their quality of life and lessen the burden.
